# An assessment of time toxicity in patients receiving [^177^Lu]Lu-PSMA-617 for metastatic castration-resistant prostate cancer

**DOI:** 10.1093/oncolo/oyag327

**Published:** 2026-08-17

**Authors:** Zachary Scharf, Miguel Muniz, Elizabeth Tchitchkan, Avina Rami, Praful Ravi, Jacob J Orme, Ali Tarhini, Heather A Jacene, Daniel Sentana-Lledo, Daniel S Childs

**Affiliations:** Department of Internal Medicine, Mayo Clinic, Rochester, MN 55905, United States; Department of Medical Oncology, Mayo Clinic, Rochester, MN 55905, United States; Department of Medical Oncology, Dana-Farber Cancer Institute, Boston, MA 02215, United States; Department of Internal Medicine, Brigham and Women’s Hospital, Boston, MA 02115, United States; Department of Medical Oncology, Dana-Farber Cancer Institute, Boston, MA 02215, United States; Department of Medical Oncology, Mayo Clinic, Rochester, MN 55905, United States; Department of Medical Oncology, Mayo Clinic, Rochester, MN 55905, United States; Department of Imaging, Dana-Farber Cancer Institute, Boston, MA 02215, United States; Department of Medical Oncology, Dana-Farber Cancer Institute, Boston, MA 02215, United States; Department of Medical Oncology, Mayo Clinic, Rochester, MN 55905, United States

**Keywords:** time toxicity, LuPSMA, radioligand, theranostics, radiopharmaceuticals

## Abstract

**Background:**

[^177^Lu]Lu-PSMA-617 (LuPSMA) improves survival and quality of life in men with metastatic castration-resistant prostate cancer but requires substantial healthcare involvement. Our analysis evaluated the time toxicity associated with LuPSMA, representing the first dedicated radioligand time toxicity assessment.

**Materials and Methods:**

We conducted a multi-institutional retrospective study of patients initiating LuPSMA between April 2022 and March 2023. Patients were followed from first LuPSMA administration until 3 months after the final cycle, initiation of new systemic therapy, or death. Time toxicity was quantified as healthcare contact days during the at-risk interval. Associations with baseline characteristics were evaluated using negative binomial regression models. Univariate and multivariable models were performed.

**Results:**

The median age of 135 patients included in the study was 69 years, and the median prior systemic therapies was 4. Patients completed a median of 5 LuPSMA cycles over 7.6 months. Median healthcare contact days were 20 (IQR, 15-28), representing 9.5% (IQR, 6.4-13.9) of at-risk days. Fifteen patients (11%) experienced high time toxicity (>1 contact day per 5 days), while 42 (31%) had low time toxicity (≤1 contact day per 14 days). Among the 57 patients who received both cycle 1 and cycle 6, the mean per cycle healthcare contact days were 4 and 3.6, respectively (paired Wilcoxon signed-rank test, *P* = .04). Routine oncology visits comprised 32.7% of contact days, compared with 8.8% from hospitalizations. Lower baseline hemoglobin was independently associated with greater time toxicity (RR = 0.87; 95% CI, 0.79-0.96; *P* = .004).

**Conclusion:**

In heavily pretreated patients, LuPSMA demonstrated relatively low time toxicity, supporting it as a time-efficient therapy.

Implications for Practice This manuscript characterizes the time toxicity associated with [177Lu]Lu-PSMA-617 (LuPSMA) therapy and represents the first dedicated assessment of time toxicity in the radioligand therapy space. It is important because it demonstrates that patients have relatively few healthcare interactions while receiving treatment and that time toxicity does not appear to vary by age. This finding distinguishes LuPSMA from traditional taxane chemotherapy, which tend to be less well tolerated in older patients. Clinically, we hope this data will better inform patient–physician discussions in the increasingly crowded treatment landscape for metastatic castration-resistant prostate cancer.

## Introduction

Usage of [^177^Lu]Lu-PSMA-617 (LuPSMA) has steadily risen since regulatory approval in March 2022.^1^^,^^2^ This medication initially showed activity in the treatment of highly refractory PSMA-positive metastatic castration-resistant prostate cancer (mCRPC), resulting in an improvement of median overall survival by around 4 months compared with a control group receiving best supportive care.^3^ The popularity of LuPSMA is driven not only by the treatment’s efficacy but also by the perception of a manageable side effect profile and a favorable dosing schedule.^4^ It is typically administered once every 6 weeks for up to 6 doses.^5^ Safety assessments, including a physical examination and laboratory studies, are performed prior to each infusion. Additionally, some institutions will use same day, or next day, SPECT imaging for treatment monitoring, but typically, no additional appointments are required until the next cycle of treatment unless the patient or provider has toxicity concerns.^6^^,^^7^ While receiving LuPSMA, some patients may have up to 6 weeks between points of healthcare contact, though others require more frequent evaluations. Data on the topic of “time toxicity” for patients receiving radiopharmaceuticals is currently limited. The concept of time toxicity has been introduced into the literature as a way to measure healthcare exposure and quantify tradeoffs in a patient-centric fashion. Definitions vary, but in general, this metric includes time spent on visits to healthcare facilities for appointments or testing, as well as for unplanned care utilization, such as urgent care visits, emergency department visits, or hospitalizations.^8^ Although relevant across all types of illnesses, the concept of time toxicity has particular importance for patients who confront care decisions in the context of a limited prognosis, such as in advanced cancer care.^9^

Quantifying time toxicity allows for the weighing of time incurred receiving cancer treatments against the anticipated survival gains from pursuing a particular therapeutic option. Previously conducted clinical trials have shown that time toxicity associated with a treatment could even surpass a potential survival benefit.^10^ In a retrospective study of patients with mCRPC during their final year of life, chemotherapy and radium-223 were associated with greater healthcare utilization than no active treatment during an early 90-day period, whereas androgen receptor pathway inhibitor (ARPI) therapy was not associated with a statistically significant increase.^11^ To our knowledge, such an investigation into time toxicity for LuPSMA has not yet been conducted, and this manuscript would represent the first dedicated time toxicity analysis for radioligand therapy in the mCRPC literature. Given the increasing use of LuPSMA, this multi-institution, real-world cohort examines and quantifies the time toxicity for patients receiving LuPSMA therapy for the treatment of mCRPC.

## Methods

### Study design and patient cohort

Each institution maintains separate IRB-approved registries (IRB IDs: Mayo 22-010856 and DF/HCC 21-629), allowing for retrospective collection of clinical outcomes data for patients with prostate cancer receiving LuPSMA. Data are de-identified at the time of abstraction to maintain confidentiality of subjects’ information. Given the retrospective, observational design, the requirement for individual study-specific consent was waived at both institutions.

The patient cohort for this study includes individuals who began LuPSMA between April 2022 and March 2023. All patients had prior exposure to an ARPI and taxane chemotherapy. During the study period, 405 patients across Mayo Clinic and Dana-Farber Cancer Institute met the study eligibility criteria. Because abstraction of time-toxicity data is highly labor-intensive, a pre-specified random sample comprising one-third of eligible patients was selected for detailed review as a pragmatic and feasibility-driven approach. Patients were randomly selected using a computer-based number generator developed for research purposes, linked to de-identified study ID number, as to not bias the sample by over-representing the experience of patients treated shortly after drug approval.^12^ This process yielded a final study cohort of 135 patients.

### Observation period

Each patient was evaluated within a defined assessment window. This time interval started on the date of the first LuPSMA treatment and continued until the earliest occurrence of one of the following: (1) 3 months after the last LuPSMA cycle, (2) initiation of a new systemic therapy, or (3) death. Data collection stopped at whichever of these criteria occurred first. This period is referred to as the “at-risk” interval.

### Definition of healthcare contact day

We retrospectively reviewed all healthcare encounters from available medical records for each patient during the at-risk interval. Dates of each healthcare contact (relative to cycle 1, day 1) were cataloged and classified by the type of contact. Each encounter was assigned to one (or more) of 8 pre-defined categories of healthcare contact: (1) hospitalization (inpatient admissions), (2) emergency department visits, (3) routine oncologic clinic visits (scheduled consultations or follow-up in oncology clinic +/− LuPSMA treatment), (4) other clinic visits (visit with another subspecialty or an unanticipated, urgent oncology visit), (5) imaging studies (visits for diagnostic imaging such as CT, MRI, SPECT, or PET scans), (6) laboratory testing (visits solely for blood draws or lab tests), (7) transfusions (outpatient visits for blood product transfusion), and (8) other contacts—a miscellaneous category for healthcare interactions not covered above (for example, radiation therapy for symptomatic bone lesions). Categorization was performed by 3 investigators (Z.S., E.T., and A.R.), and any ambiguities were resolved by consensus.

In cases where multiple types of services were provided in a single day (for instance, a clinic visit plus same-day lab tests and imaging), each service was recorded, but for analysis purposes, this was counted as a single healthcare contact day. We chose to aggregate same-day services into a single contact day on the assumption that multiple services clustered on one day represent a lower cumulative burden of time and travel for the patient compared to those services spread over multiple days. Such assumptions are standard in time toxicity literature.^10^^,^^13^

Home days were those when patients had no in-person contact with healthcare services. Telemedicine appointments and home care visits were not counted toward healthcare contact days.

### Statistical analysis/methods

The primary outcome measure for time toxicity was the percentage of healthcare contact days per patient over the at-risk interval. This was calculated as the total number of contact days divided by the total number of days in that patient’s at-risk interval. This fraction represents the portion of days during follow-up on which the patient physically interacted with the healthcare system. We summarized the distribution of these fractions across the cohort to evaluate variability in time burden between patients, reporting the percentage of patients spending more than 20% (>1 in 5) of at-risk days in contact with the healthcare system and that of patients spending less than or equal to 1 day in 14 interacting with the healthcare system. These cutoffs have been utilized in prior time toxicity literature.^14^ We also report median healthcare contact days by cycle to illustrate how the burden of time toxicity changes with each cycle of treatment. The frequency and relative proportion of each type of healthcare contact were calculated for the entire cohort (eg, the percentage of all contact days that involved emergency department visits). Descriptive statistics were used to characterize patterns of healthcare utilization.

Cycle-specific healthcare contact days were summarized descriptively among patients reaching each treatment cycle. Healthcare contact days during cycle 1 and cycle 6 were compared using a paired Wilcoxon signed-rank test restricted to patients who received both cycles. Associations between baseline characteristics and contact rates were assessed using negative binomial regression with an offset for the at-risk duration, selected to account for count-based and over-dispersed outcome data. Univariate and multivariable models were performed, including the core variables of creatinine, hemoglobin, age, and the distribution of metastatic disease (i.e. visceral metastases), which draw upon prior studies as hypothesized or reported predictors of time toxicity in other work.^15–17^ Baseline data for all covariates included in the regression models were available for all 135 patients; therefore, no patients were excluded because of missing covariate data, and no imputation was required. A sensitivity analysis was performed to ensure that pooling the data between the 2 sites is appropriate. Statistical analyses were conducted using BlueSky Statistics Version 10.3.4 and R package version 8.95. Graphical representations were generated with Excel and BioRender.

## Results

**Figure 1.**
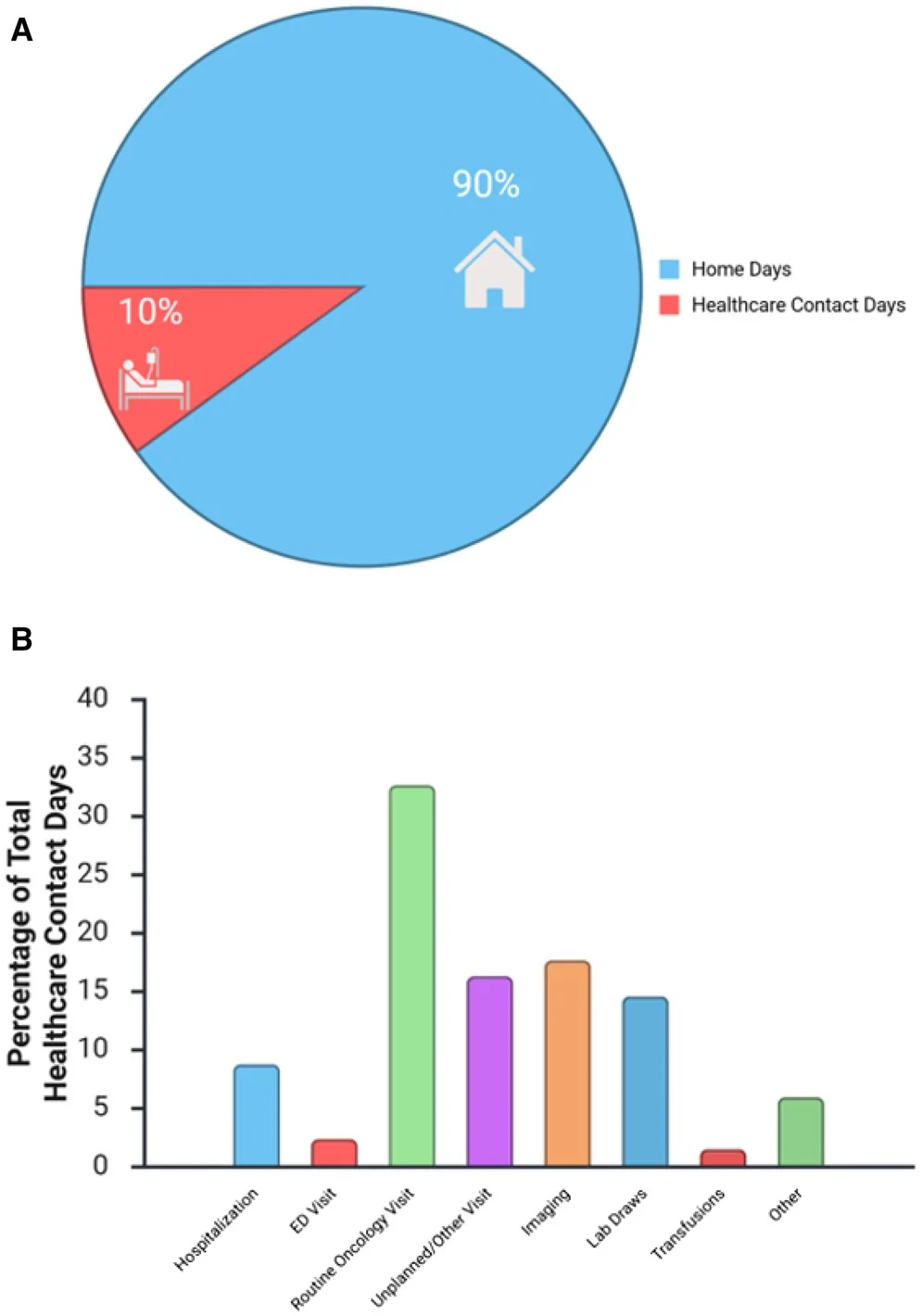
Proportion of days with healthcare encounters (A) and associated visit reasons (B). (A) The percentage of days at home vs interacting with the healthcare system for the cohort. (B) Each line represents a unique reason for healthcare contact.

The cohort included 135 patients from 2 cancer centers who began LuPSMA between April 2022 and March 2023, when it was approved only for patients with mCRPC progressing after ARPIs and taxane chemotherapy. The baseline characteristics are presented in Table 1. We highlight a median age at the start of LuPSMA therapy of 69 years (range 45-88), median number of prior therapies of 4 (range, 2-9), and 43 patients (32%) with visceral metastases at the start of LuPSMA. Table 2 illustrates LuPSMA treatment details with patients receiving a median of 5 cycles (IQR, 3-6) over a duration of 7.6 months (IQR, 5.2-10.1). During the at-risk period, patients had a median of 20 healthcare contact days (IQR 15-28), representing 9.5% (IQR 6.4-13.9) of total at-risk days (Figure 1). Median healthcare contact days as a percentage of total at-risk days were nearly identical between the Mayo (9.58%) and Dana-Farber Cancer Institute (9.52%) sites, with no statistical difference observed (Wilcoxon *P* = .84). Routine oncology visits were the most common reason for healthcare contact (32.7%), followed by imaging appointments (17.7%). A total of 43 (32%) and 46 (34%) patients had an emergency department visit or hospitalization during the at-risk interval, accounting for 2.4% and 8.8% of the total contact days, respectively.

**Table 1.** Baseline characteristics.

Characteristics	Value
**Median age at start of LuPSMA, years (range)**	69 (45-88)
**Race/ethnicity, *n* (%)**	
** Caucasian**	122 (90.4)
** African American**	3 (2.2)
** Asian**	4 (3.0)
** Hispanic**	3 (2.2)
** Other/chose not to disclose**	3 (2.2)
**Metastatic distribution, *n* (%)**	
** Lymph node**	92 (68.2)
** Bone**	119 (88.2)
** Visceral/other**	43 (31.9)
**Median time from diagnosis to first LuPSMA, months (range)**	56.9 (6.5-309.1)
**Baseline laboratory parameters at start of LuPSMA (IQR)**	
** Median PSA, ng/mL**	17.7 (2.0-143.3)
** Median hemoglobin, g/dL**	11.7 (10.2-13.1)
** Median platelets × 10^9^/L**	209 (172.5-254)
** Median white blood cells**	5.5 (4.2-6.9)
** Median creatinine**	1.0 (0.8-1.1)
**Median number of prior lines of systemic therapy, *n* (range)**	4 (2-9)

Abbreviations: IQR, interquartile range 25%-75% percentiles; LuPSMA, [^177^Lu]Lu-PSMA-617.

**Table 2.** LuPSMA treatment characteristics.

Characteristics	LuPSMA (*n* = 135)
**Median duration of at-risk interval, months (range)**	7.6 (0.2-11.9)
**Total number of LuPSMA cycles, *n* (%)**	
** 1**	9 (6.7)
** 2**	17 (12.6)
** 3**	24 (17.8.5)
** 4**	14 (10.4)
** 5**	14 (10.4)
** 6**	57 (42.2)
**Median number of cycles of LuPSMA (IQR)**	5 (3-6)

Abbreviations: IQR, interquartile range 25%-75% percentiles; LuPSMA, [^177^Lu]Lu-PSMA-617.

**Figure 2.**
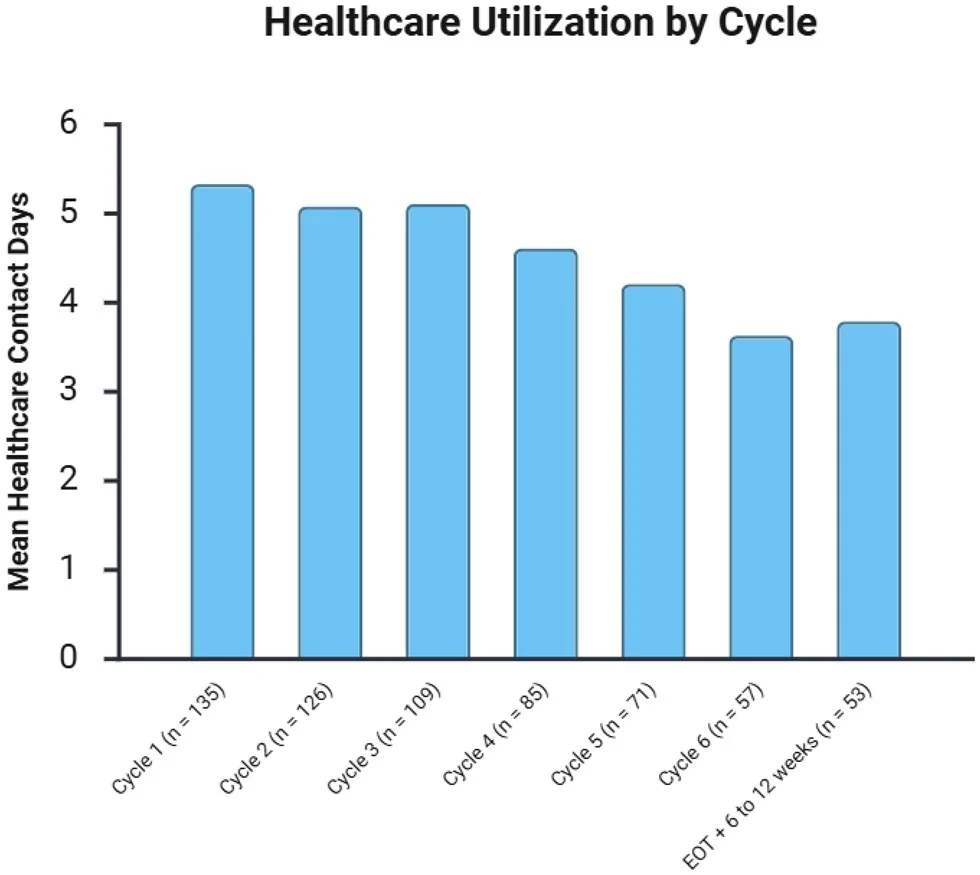
Mean healthcare contact days by cycle. Mean healthcare contact days are presented descriptively among patients reaching each cycle. The number of evaluable patients decreased across successive cycles. A paired cycle 1 vs cycle 6 comparison that was restricted to the 57 patients who received both cycles and is reported in the text. EOT, end of treatment.

**Figure 3.**
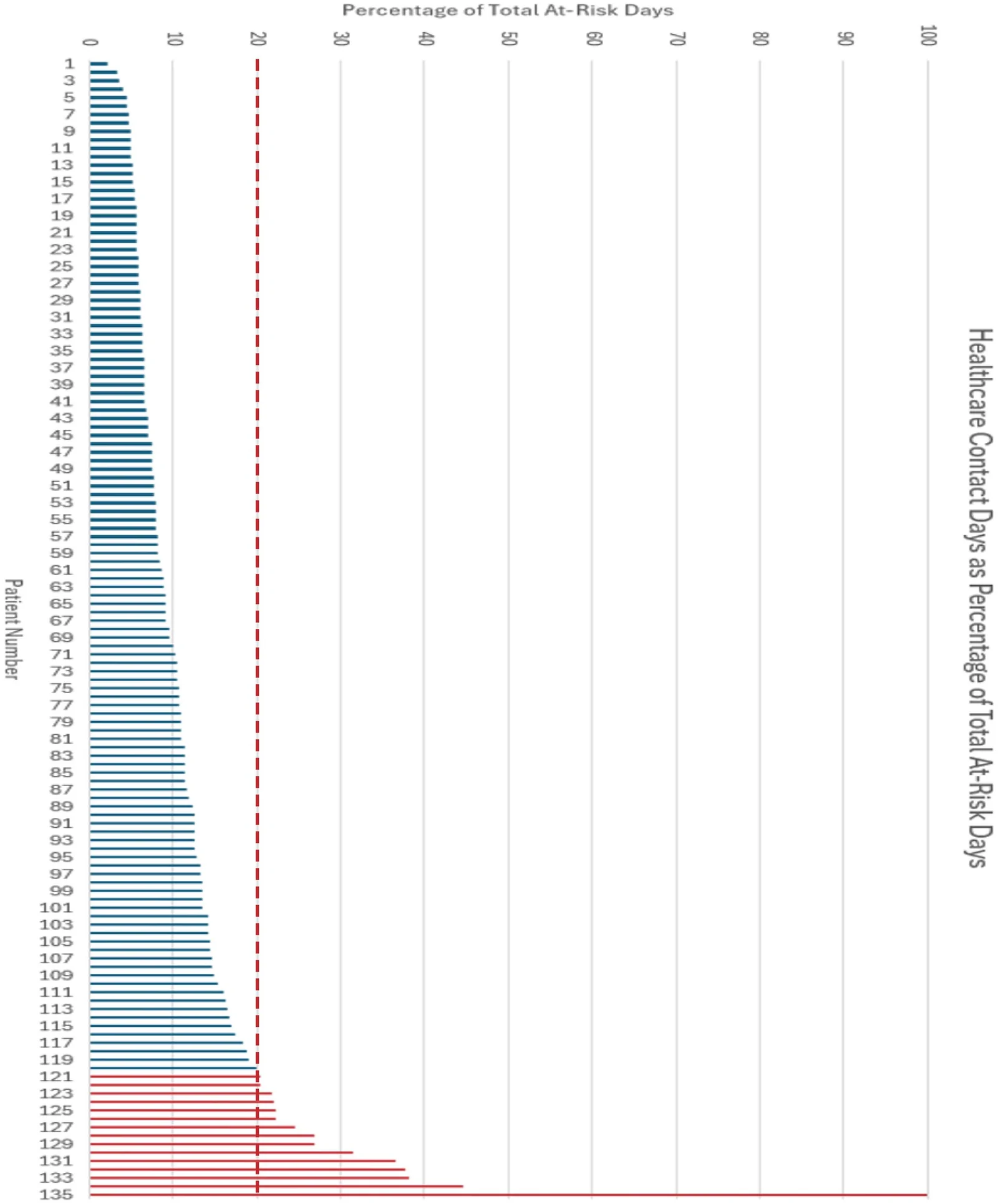
Patient-level distribution: percentage of at-risk days with healthcare contact. Each line represents an individual patient in the cohort and charts their percent of healthcare contact.

Figure 2 descriptively depicts the average number of healthcare contact days among patients reaching each treatment cycle. The number of evaluable patients decreased from 135 during cycle 1 to 57 during cycle 6. Among the 57 patients who received both cycles, mean healthcare contact days were 4 during cycle 1 and 3.6 during cycle 6 (paired Wilcoxon signed-rank test, *P* = .04). Variability in the number of healthcare contact days among individual patients is illustrated in Figure 3. Only 15 patients (11%) exhibited categorically high time toxicity, which was defined as spending more than 20% (Healthcare Contact Days/Total At-Risk Days) of their at-risk days engaged with the healthcare system. In contrast, low time toxicity (≤1 day out of 14) was observed in 42 patients (31%).

Associations between baseline patient or disease characteristics and the risk for time toxicity were assessed. Core variables of creatinine, hemoglobin, age, and the distribution of metastatic disease (i.e. visceral metastases) were included in the univariate and multivariable analyses (Table 3). Lower baseline hemoglobin was associated with higher time toxicity (RR = 0.87; 95% CI, 0.79-0.96; *P* = .004). Visceral metastases were associated with increased healthcare utilization on univariate analysis, but this association was not statistically significant on multivariable analysis (RR = 1.35; 95% CI, 0.92-2; *P* = .13). Creatinine and age were not found to have significant associations with time toxicity.

**Table 3.** Univariate and multivariable analyses of associations between clinical factors and healthcare contact days.

	Rate ratio	95% CI low	95% CI high	*P*-value
**Univariate analyses**				
** Hemoglobin**	0.86	0.78	0.94	.001
** Visceral metastases**	1.50	1.02	2.19	.038
** Creatinine**	0.97	0.52	1.80	.92
** Age**	0.99	0.97	1.02	.60
**Multivariate analyses**				
** Hemoglobin**	0.87	0.79	0.96	.004
** Visceral metastases**	1.35	0.92	2.01	.13
** Creatinine**	0.96	0.51	1.79	.89
** Age**	0.99	0.97	1.01	.37

## Discussion

It is well known that quality of life is worsened by the considerable time commitments required for cancer care.^16^^,^^17^^,^^18,1,9^ One would expect LuPSMA to have a favorable time toxicity profile given it involves infrequent treatments (once every 6 weeks) with short intravenous infusions; however, clinical data from the ambulatory setting to this point has been lacking. Our findings show that patients treated with LuPSMA spent nearly 90% of days at home without any form of healthcare contact, supporting the prevailing perception that this particular treatment strikes a favorable balance between efficacy and time demands. When combined with quality-of-life findings from VISION and PSMAfore, these results reinforce the overall tolerability of LuPSMA, demonstrating that patients can preserve their well-being while spending time away from healthcare environments.^20^^,^^21^

Though most patients had minimal interaction with the healthcare system during treatment, approximately 10% of patients receiving LuPSMA had a much higher contact frequency (>1 in every 5 days). Lower baseline hemoglobin was associated with increased number of contact days. This association may reflect underlying disease burden and prognosis as baseline anemia is a well-established prognostic variable for overall survival in patients receiving LuPSMA^15^^,^^22^ and cumulative days spent on healthcare contact typically increases for patients with more guarded prognoses.^11^ Nevertheless, baseline hemoglobin may serve as a readily available marker to help identify patients who could benefit from additional support or intensified remote monitoring during LuPSMA treatment.

Notably, increasing age did not predict higher healthcare utilization during treatment with LuPSMA. Others have reported that older adults (>65 years) with metastatic cancer spend up to 1 in 5 days in contact with the healthcare system, and the burden is even higher when receiving cytotoxic chemotherapy.^9^ Particularly in the last quarter of life, the burden rises, driven largely by emergency room visits and hospitalizations.^11^ For LuPSMA, the absence of an age effect on time toxicity may represent an important distinguishing feature for patients trying to decide between treatment with taxane-based chemotherapy versus a radiopharmaceutical.

A final notable observation is that roughly 1 in 3 patients receiving LuPSMA will have an emergency department visit or hospitalization during the treatment period. While emergency department visits or hospital admissions were common, the duration of stay was typically short, with these encounters accounting for about 11% of the total healthcare contact days. Our cohort of patients had highly treatment-refractory disease with a median of 4 prior lines of therapy, resembling the VISION trial population. It is reasonable to expect that unplanned care utilization would be lower when LuPSMA is administered earlier in the disease course such as in the pre-taxane or hormone sensitive settings.

Several limitations should be acknowledged. First, healthcare contact days were abstracted from electronic medical records rather than patient self-report, which may underestimate burden for those receiving care at locations outside our record system. Second, institutional protocols vary with some centers performing next-day SPECT imaging or mid-cycle labs, which may inflate or reduce contact day counts. Third, the retrospective design limited accurate assessment of other important factors such as frailty, comorbidity burden, and social determinants of health, which have been associated with higher time toxicity. More granular time metrics like travel length from home or duration of appointments in minutes or hours were not feasible. Additionally, the comparison between cycle 1 and cycle 6 was restricted to patients who received all 6 cycles and is therefore susceptible to survivor and treatment-selection bias. Patients reaching cycle 6 were more likely to have survived, tolerated treatment, and maintained sufficient disease control to continue therapy. Consequently, the modestly lower number of healthcare contact days observed at cycle 6 within this subgroup should not be interpreted as demonstrating that time toxicity decreases with continued LuPSMA treatment. A particular strength of this analysis is that it includes a large, multi-center dataset which captures different institutional practices regarding LuPSMA administration and monitoring, and supports applicability in different practice settings. However, the predominantly white composition of the cohort should be considered when extending these findings to more ethnically diverse populations. To our knowledge, only one other study has touched upon the topic of time toxicity in patients receiving LuPSMA; however, it included only one patient on this treatment regimen, illustrating a void in the literature.^11^

Taken together, these findings underscore that LuPSMA is not only safe and effective, but it has a generally favorable time toxicity profile. This attribute makes it an attractive choice for patients with mCRPC who prioritize their time away from clinical settings and seek to maintain quality of life during therapy.

## Conclusion

Only about 10% of days during treatment with LuPSMA involve physical contact with the health care system. A favorable dosing schedule, the limited treatment monitoring requirements, and general tolerability afford patients more time and freedom from frequent points of healthcare contact.

## Data Availability

The authors confirm that the data supporting the findings of this study are available within the article. Raw data that support the findings of this study are available from the corresponding author, upon reasonable request.
